# THE ROAD MAP STRATEGIST INITIATIVE: A NATIONWIDE EFFORT TO BUILD LOCAL PUBLIC HEALTH CAPACITY ON DEMENTIA

**DOI:** 10.1093/geroni/igac059.1766

**Published:** 2022-12-20

**Authors:** Meghan Fadel, Peter Holtgrave, Shelby Roberts, Eva Jackson, John Shean, Lisa Garbarino, Lisa McGuire

**Affiliations:** Alzheimer's Association, Albany, New York, United States; National Association of County and City Health Officials (NACCHO), Washington, District of Columbia, United States; Alzheimer's Association, Chicago, Illinois, United States; Alzheimer's Association, Chicago, Illinois, United States; Alzheimer's Association, Chicago, Illinois, United States; Centers for Disease Control and Prevention, Atlanta, Georgia, United States; CDC, Atlanta, Georgia, United States

## Abstract

The Road Map Strategist Initiative is a nationwide effort to build local health department (LHD) capacity to address cognitive health and dementia. Through a collaboration between the Alzheimer’s Association and the National Association of County and City Health Officials (NACCHO), the initiative aims to make policy, systems, and environmental change, with a focus on health equity. Participating health departments receive funding, training, and technical assistance to establish a part-time Healthy Brain Initiative (HBI) Road Map Strategist, a public health professional serving as a system change agent. With support and education from the program sponsors, Road Map Strategists conduct a community health needs assessment, train LHD staff and leadership, and collaborate with community partners to implement and accelerate public health actions on dementia. Actions taken by the Road Map Strategists are informed by the Healthy Brain Initiative’s State and Local Public Health Partnerships to Address Dementia, The 2018-2023 Road Map. Presenters will share lessons learned from the first cohort of eight (8) LHDs during each stage of the initiative, with a focus on approaches used for Road Map implementation efforts in their communities. This session will share evaluation findings on successes and barriers for changing health department capacity to support local dementia efforts. Data from key indicators related to knowledge gain and capacity change will be shared, including the way LHD characteristics influenced success. The session will also cover effective approaches toward advancing health equity in dementia in local communities and plans for future iterations of the initiative.

